# Glucose Tolerance Tests and Osteocalcin Responses in Healthy People

**DOI:** 10.3389/fendo.2018.00356

**Published:** 2018-07-13

**Authors:** Jakob Starup-Linde, Sidse Westberg-Rasmussen, Simon Lykkeboe, Aase Handberg, Bolette Hartmann, Jens J. Holst, Kjeld Hermansen, Peter Vestergaard, Søren Gregersen

**Affiliations:** ^1^Department of Endocrinology and Internal Medicine, Aarhus University Hospital, Aarhus, Denmark; ^2^Department of Clinical Biochemistry, Aalborg University Hospital, Aalborg, Denmark; ^3^Department of Biomedical Sciences and The NNF Center for Basic Metabolic Research, University of Copenhagen, Copenhagen, Denmark; ^4^Departments of Clinical Medicine and Endocrinology, Aalborg University Hospital, Aalborg, Denmark; ^5^Steno Diabetes Center North Jutland, Aalborg, Denmark

**Keywords:** osteocalcin, undercarboxylated osteocalcin, glucose, insulin, oral glucose tolerance test, isoglycemic intravenous glucose infusion

## Abstract

**Aim:** Osteocalcin and undercarboxylated osteocalcin are suggested to be endocrine messengers from the bones and have been shown to stimulate insulin secretion from pancreatic β-cells. Insulin is hypothesized to increase the osteoblastic production of osteocalcin. The aim of the study was to investigate whether the route of glucose administration influence the circulating levels of osteocalcin and undercarboxylated osteocalcin.

**Methods**: Twelve healthy males were enrolled in an acute cross-over study where they underwent an oral glucose tolerance test (OGTT), an isoglycemic intravenous glucose infusion (IIGI) and a fasting period (control). Blood samples were collected throughout 180 min and analyzed for osteocalcin and undercarboxylated osteocalcin and compared to insulin, glucose, and gastro-intestinal hormone responses.

**Results:** Neither osteocalcin levels nor undercarboxylated osteocalcin levels over time differed between the OGTT, IIGI, and fasting. Baseline insulin levels and glucose levels were not associated with osteocalcin or undercarboxylated osteocalcin levels. Increases in insulin and glucose levels were neither associated with altered osteocalcin nor undercarboxylated osteocalcin levels.

**Conclusion:** The route of glucose administration does not influence the circulating levels of osteocalcin and undercarboxylated osteocalcin despite the differential insulin and incretin responses. In the acute setting this suggests that insulin does not increase osteoblastic production of osteocalcin in healthy human males.

## Introduction

Osteocalcin is suggested to be an endocrine product and messengers from the bones. Osteocalcin is produced by the osteoblasts and becomes undercarboxylated through the acidification in relation to bone resorption (1). In animal models undercarboxylated osteocalcin is proposed to increase insulin production and release by a direct effect on pancreatic β-cells (1–3). Conversely, insulin is believed to affect the osteoblasts directly and to increase osteocalcin production (1). Thus, a positive feedback-cycle where osteocalcin and undercarboxylated osteocalcin modulate glucose metabolism has been proposed to exist (1). In humans osteocalcin and undercarboxylated osteocalcin are inversely associated with fasting plasma glucose and glycated hemoglobin A1c supporting the hypothesis of a bone pancreas-axis (4). Furthermore, postmenopausal osteoporotic females treated with parathyroid hormone (PTH) have increased circulating osteocalcin and undercarboxylated osteocalcin levels and decrease blood glucose levels during an oral glucose tolerance test (5). However, in hypoparathyroid individuals treated with PTH an increase in undercarboxylated osteocalcin does not affect fasting plasma glucose or insulin resistance (6).

An oral glucose tolerance test (OGTT) and an intravenous isoglycemic glucose infusion (IIGI) results in differential insulin and incretin hormone responses despite comparable levels of plasma glucose (7, 8). The aim of the present study is to investigate whether administration of glucose by two different routes resulting in differential incretin and insulin responses differentially influences osteocalcin and undercarboxylated osteocalcin levels.

## Patients and methods

The study was registered at ClinicalTrials.gov (NCT02213276). Approval was obtained from the Danish Data Protection Agency (2007-58-0010) and the Ethics Committee of the Central Denmark Region (1-16-02-377-13) and relevant guidelines were followed. All subjects gave written informed consent in accordance with the Declaration of Helsinki. The STROBE guidelines were followed (Supplemental Table 1). The study design has been described previously in detail (8). In the study we included relatively young individuals and to avoid potential effects of fluctuations in sex hormones on bone markers during the menstrual cycle we only enrolled males. Briefly, 12 healthy Caucasian males, aged 20 to 50 years, were included in the study and underwent an OGTT and an IIGI. Eight of the participants also engaged in a fasting control. During the OGTT participants drank a glucose solution consisting of 82.5 g of glucose monohydrate (equal to 75 g of D-glucose), 225 ml of water and 225 mg of benzoic acid over 5 min. For the IIGI 20% D-glucose was infused in the antecubital vein and mimicked the glucose levels of the OGTT by adjusting the infusion rate for the glucose solution according to the plasma glucose level. Blood samples were collected at −15, −10, 0, 15, 30, 60, 120, and 180 min from initiation of the glucose administration at the OGTT and IIGI and plasma glucose levels were measured every 5 min. A fasting control study (without glucose administration) was also performed where blood samples were collected after 0, 1, 2, and 3 h. The day before each intervention, participants were asked to refrain from exercise, smoking and taking vitamin supplements. A standard meal delivered by the clinic was to be ingested between 17 and 23 o'clock and participants were asked to fast (water allowed) from 23 o'clock until they arrived for the intervention the next morning. They were asked to arrive by car or bus to the clinic.

### Biochemical measures

Manufacturer determined standard deviations for intermediate precision is 0.1 μM, within a glucose concentration of 2.5–6.5 μM. Serum levels of osteocalcin were measured on an automated Cobas E601 analyzer (Roche Diagnostics, Mannheim, Germany) and quality control material parallelly measured with test samples resulted in variation coefficients less than 1%. EDTA-plasma levels of undercarboxylated osteocalcin were measured by ELISA (Takara Bio Inc, Otsu, Japan) and samples from each participant were analyzed within a single ELISA-plate. Within plate precision judged from duplicate measurements attained variation coefficients less than 4%. Intermediate precision determined by analyzing quality control material resulted in variation coefficients less than 10%.

Analysis of and data on plasma glucose, plasma insulin, glucagon-like peptide-1 (GLP-1), glucagon-like peptide-2 (GLP-2), and gastric inhibitory peptide (GIP) have previously been described (8).

Briefly, plasma glucose was measured using the Accu-Chek Inform II apparatus (Roche Diagnostics, Basel, Switzerland) and plasma insulin was measured by ELISA using a DAKO insulin kit (Code: K6219; Dako, Glostrup, Denmark).

### Statistical analysis

Statistical analysis was carried out using the STATA 14 package (StataCorp, College Station, Texas, USA). Repeated measures ANOVA was performed to compare levels of osteocalcin, undercarboxylated osteocalcin, insulin, glucose, and gastrointestinal hormones between OGTT, IIGI and the 3-h fasting control. Normality of data was checked via qq-plots. The compound symmetry assumption was checked by examining the pooled within-subject covariance. We performed three conservative *F*-tests to ensure validity of results.

Using a simple linear regression model, the association between the increase in insulin, GIP, GLP-1, GLP-2, and glucose from 0 to 30 min and alterations in osteocalcin and undercarboxylated osteocalcin during same time period were examined. Furthermore, the association between baseline osteocalcin, undercarboxylated osteocalcin and insulin and glucose were analyzed by simple linear regression. No power calculation was performed on osteocalcin or undercarboxylated osteocalcin Osteocalcin measurement was a secondary outcome measured subsequent to the publication of the main findings. This was performed in order to gain further knowledge. The primary endpoint of the study was CTX which is previously published (8).

## Results

**Figure 1:**
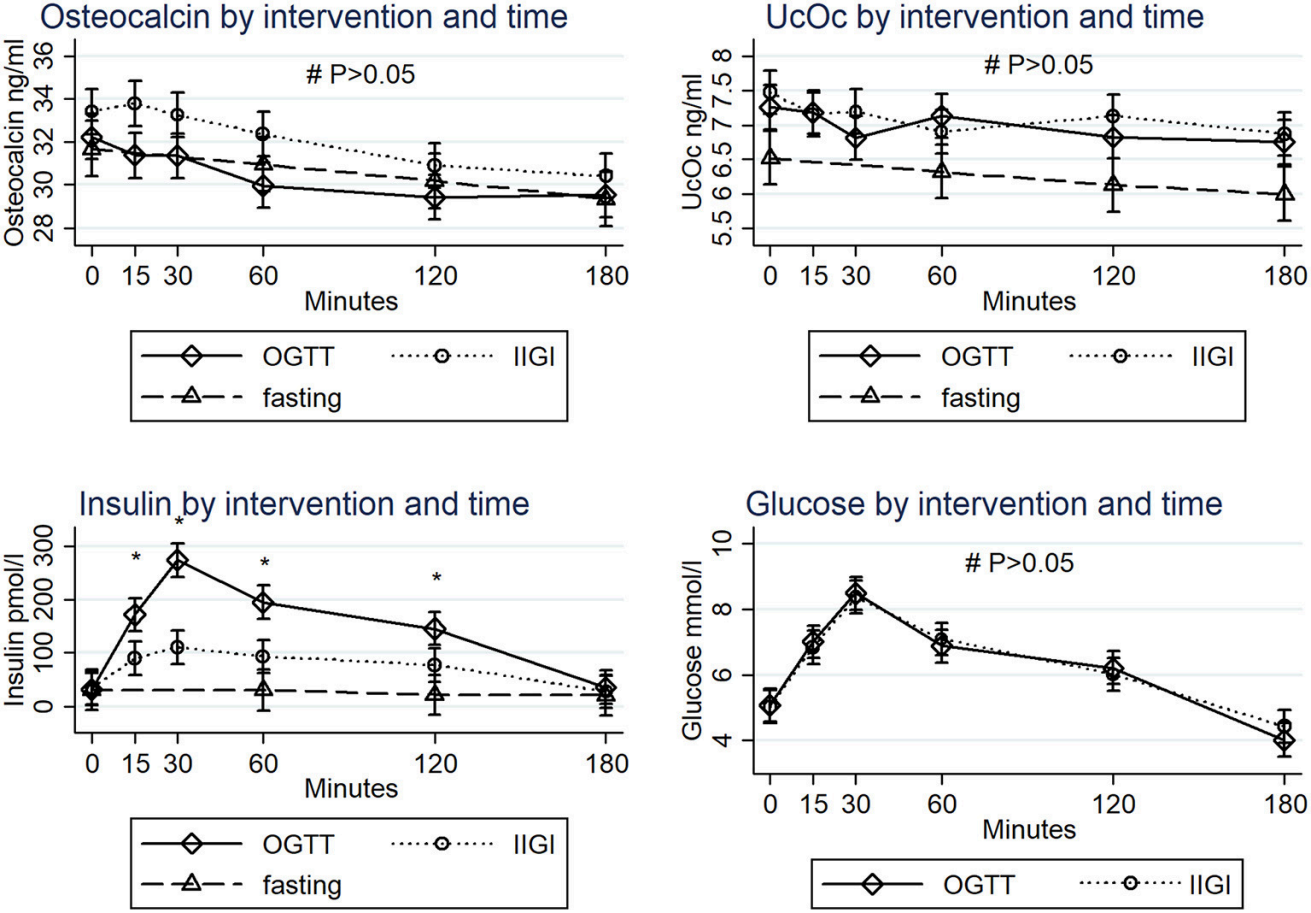
Plasma osteocalcin, undercarboxylated osteocalcin, insulin, and glucose responses to oral glucose tolerance test (OGTT), intravenous isoglycemic glucose infusion (IIGI) and fasting by time. ^*^Significantly different between OGTT, IIGI and fasting (*P* < 0.05). **#** No difference was detected between OGTT, IIGI, and fasting (*P* > 0.05). The error bars represent 95% confidence intervals. Undercarboxylated osteocalcin (UcOc).

The mean age of the healthy participants was 30.6 years and the mean body mass index was 24.2 kg/m^2^. All participants presented with normal HbA1c. The participant characteristics have previously been described. Figure 1 presents the responses of osteocalcin, undercarboxylated osteocalcin, insulin, and glucose by time and intervention.

As intended, the plasma glucose curves levels were comparable during the OGTT and IIGI. The insulin concentrations increased during the OGTT and IIGI with peak values at 30 min (274 vs. 32 pM and 111 vs. 34 pM, respectively). No changes in insulin or glucose concentrations were observed during fasting.

Baseline levels of osteocalcin were 32.2 ± 9.3, 33.4 ± 8.5, and 31.7 ± 8.3 ng/ml at the OGTT, IIGI, and fasting interventions, respectively. Baseline levels of undercarboxylated osteocalcin were 7.3 ± 3.2, 7.5 ± 2.8, and 6.5 ± 2.3 ng/ml at the OGTT, IIGI, and fasting interventions, respectively. No interaction was observed between time and type of intervention for osteocalcin and undercarboxylated osteocalcin (*P* > 0.05). Osteocalcin and undercarboxylated osteocalcin levels were not significantly different at any time points between OGTT, IIGI, and fasting (*P* > 0.05).

During the OGTT osteocalcin decreased significantly at 60 min (30.0 ± 8.4 ng/ml), 120 min (29.4 ± 8.1 ng/ml), and 180 min (29.6 ± 8.2 ng/ml) compared to baseline. During the IIGI osteocalcin decreased significantly at 120 min (30.9 ± 7.2 ng/ml) and 180 min (30.4 ± 7.3 ng/ml) compared to baseline whereas fasting only decreased osteocalcin at 180 min (29 ± 7.2 ng/ml) compared to baseline.

Undercarboxylated osteocalcin decreased significantly at 180 min (6.7 ± 2.8 ng/ml) during the OGTT and at 60 min (6.9 ± 2.6 ng/ml) and 180 min (6.9 ± 2.6 ng/ml) during the IIGI compared to baseline. No difference in undercarboxylated osteocalcin was observed over time during fasting.

Simple linear regression showed no association between baseline osteocalcin or undercarboxylated osteocalcin and glucose or insulin (*P* > 0.05). Furthermore, no association was observed between alterations in osteocalcin or undercarboxylated osteocalcin and increases in insulin, glucose, GIP, GLP-1, and GLP-2 from baseline to 30 min (*P* > 0.05).

## Discussion

In the present study we observed no increase in osteocalcin or undercarboxylated osteocalcin in response to glucose administration either orally or intravenously although these tests resulted in widely different insulin responses. Both during the OGTT and IIGI osteocalcin seemed to decrease over time. The results do not support the hypothesis of an increased osteocalcin production due to osteoblastic activation by insulin, at least in the acute setting in metabolically healthy people.

Our results corroborate previous human data. Insulin infused at high, intermediate and low doses neither affected the level of undercarboxylated osteocalcin in participants with type 2 diabetes nor in healthy individuals (9). Furthermore, OGTT has previously been shown to decrease osteocalcin as well as undercarboxylated osteocalcin (10, 11) and osteocalcin levels decreased during a hyperinsulinemic, euglycemic clamp in obese people (12). Antiresorptive therapies are hypothesized to increase undercarboxylated osteocalcin and thereby decrease diabetes incidence, however, in a *post-hoc* analysis of three randomized controlled trials the antiresorptive therapy did not affect fasting plasma glucose, weight loss, or diabetes risk (13).

Current human data do not support a causal relationship between increased insulin levels leading to increased osteocalcin and undercarboxylated osteocalcin levels. However, delta-like 1 (DLK1) that is co-localized with insulin in the pancreatic β-cells may affect these bone formation markers. DLK1 deficient mice expressed high levels of osteocalcin, whereas mice overexpressing DLK1 had low levels of osteocalcin (14). DLK1 may therefore counteract the positive feedback loop between osteocalcin and endogenous insulin (14).

The reduction in osteocalcin observed during the OGTT was comparable to that observed during the fasting. Thus there was a time-related decline during the interventions. During OGTT, both bone resorption markers and bone formation markers decrease in 20 min, however, this effect is abolished by the somatostatin analog octreotide, which inhibits the release of gastrointestinal hormones (15). In the present study osteocalcin and undercarboxylated osteocalcin were not related to circulating levels of gastro-intestinal hormones. The discrepancy compared with previous studies may be due to differences in subject characteristics.

In people with diabetes, osteocalcin levels are lower than in non-diabetic controls and the osteocalcin levels are negatively associated with glucose (16, 17). This may explain the association found in the meta-analysis by Liu et al. (4). It is likely to find an inverse association between osteocalcin and fasting plasma glucose in people with and without diabetes. Thus, fasting people with diabetes have lower levels of osteocalcin, higher levels of glucose and lower levels of insulin.

This study was a controlled experiment. However, it was not possible to blind the participants. Furthermore, the results may be different in metabolic dysregulated individuals and in women. The sample size of the study is limited and the follow-up time is relatively short which may have influenced the results.

In conclusion, we observed no differences in osteocalcin or undercarboxylated osteocalcin responses glucose challenges resulting in widely different endogenous insulin and incretin responses. Our results suggest that insulin does not increase osteoblastic production of osteocalcin in healthy humans.

## Author contributions

JS-L, SW-R, and SG performed the clinical study. JS-L and SW-R researched the data. SL and AH measured bone turnover markers. JH and BH measured incretin hormones. JS-L, SW-R, SL, AH, BH, JH, KH, PV, and SG wrote and revised the manuscript.

### Conflict of interest statement

The authors declare that the research was conducted in the absence of any commercial or financial relationships that could be construed as a potential conflict of interest.
